# Cdc48: A Swiss Army Knife of Cell Biology

**DOI:** 10.1155/2013/183421

**Published:** 2013-09-15

**Authors:** Guem Hee Baek, Haili Cheng, Vitnary Choe, Xin Bao, Jia Shao, Shiwen Luo, Hai Rao

**Affiliations:** ^1^Department of Molecular Medicine, University of Texas Health Science Center, San Antonio, TX 78229, USA; ^2^The First Affiliated Hospital, Nanchang University, Nanchang, Jiangxi 330006, China

## Abstract

Cdc48 (also called VCP and p97) is an abundant protein that plays essential regulatory functions in a broad array of cellular processes. Working with various cofactors, Cdc48 utilizes its ATPase activity to promote the assembly and disassembly of protein complexes. Here, we review key biological functions and regulation of Cdc48 in ubiquitin-related events. Given the broad employment of Cdc48 in cell biology and its intimate ties to human diseases (e.g., amyotrophic lateral sclerosis), studies of Cdc48 will bring significant insights into the mechanism and function of ubiquitin in health and diseases.

## 1. Introduction

**Figure 1:**
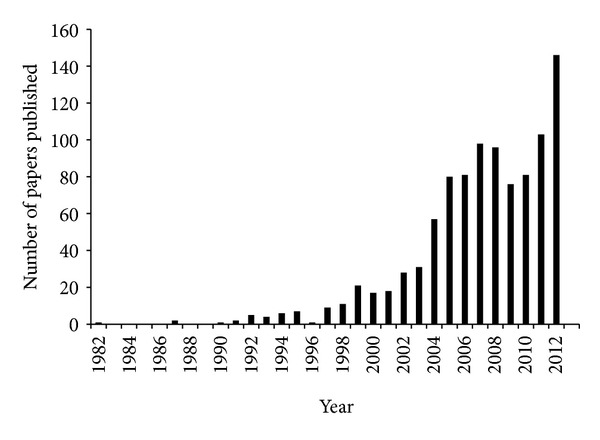
Number of papers on Cdc48 published since 1982. Key words “Cdc48, VCP, and p97” were used to search PUBMED for papers on Cdc48 from 1982 to 2012.

Thirty years ago when David Botstein's laboratory first isolated *cdc48-1* allele among 18 cold-sensitive yeast mutants defective in cell cycle progression [1], little did they know how much power is embedded in *CDC48*. Although Cdc48 was initially suspected to play a pivotal role in some nuclear event(s) essential for cell cycle progression, it is now known to be a key regulator for a myriad of cellular processes in nucleus, cytosol, mitochondria, peroxisome, endoplasmic reticulum (ER), Golgi, lysosome, and plasma membrane [2–6]. Befittingly, Cdc48 is one of the most abundant proteins in eukaryotic cells, accounting for 1% of cytosolic proteins [5, 7]. The mammalian homologue of Cdc48 is also called p97 for molecular weight or VCP (valosin-containing protein) by the groups that identified it with different approaches in various systems [1, 8, 9]. Although little attention was paid to Cdc48 early on, its stock has risen dramatically in recent years (Figure 1).

Evolutionally conserved Cdc48 is an ATPase essential for cell growth and survival. It is a central component in many ubiquitin-mediated pathways and participates in a wide range of biological events, including cell cycle regulation, protein degradation, membrane fusion, DNA replication, gene expression, DNA damage response, apoptosis, and autophagy [2–5, 7, 10, 11]. A book would be needed to comprehensively cover the various functions of Cdc48. Here, we specifically highlight a few key aspects of Cdc48: the biochemical properties, physiological regulation, and crucial biological functions.

## 2. Biochemical Properties

### 2.1. ATPase Activity

**Figure 2:**
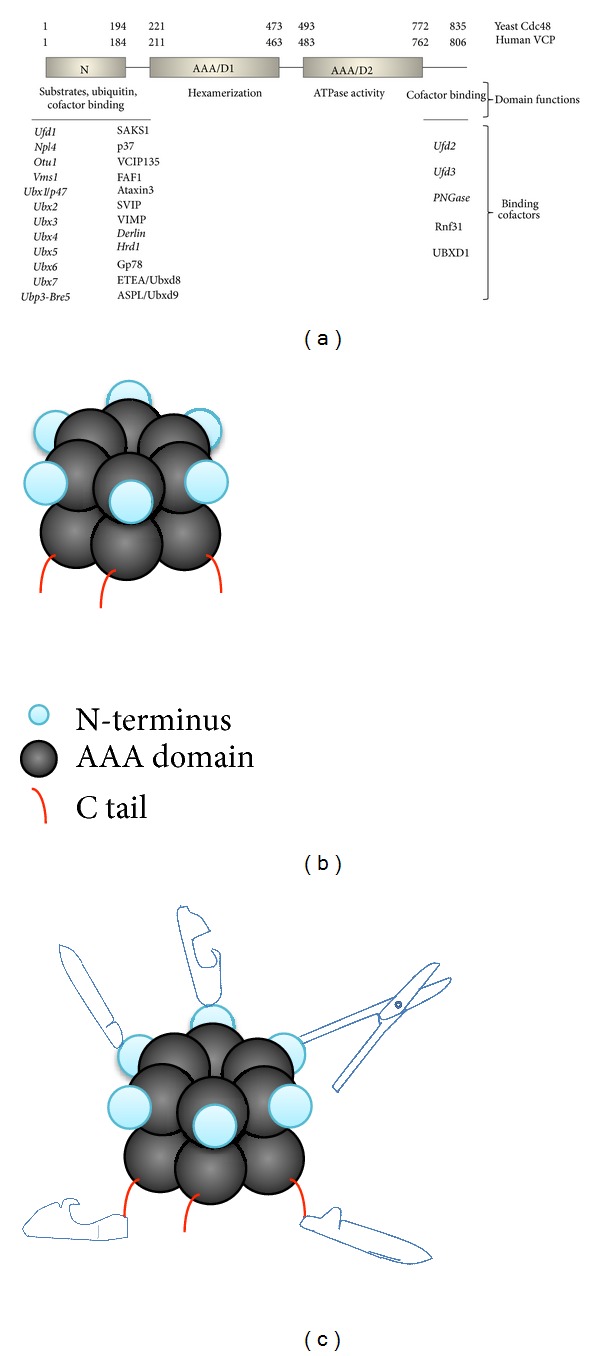
Structural and functional organization of Cdc48. (a) Cdc48 consists of the conserved N-terminal domain (N), two conserved AAA ATPase domains (D1 and D2), and the C-terminal domain (C). The numbers refer to the amino acid positions within the protein in yeast and human Cdc48, respectively. Cofactors associated with the N and C domains are listed. Yeast proteins, nearly all of which have human homologues, are highlighted in italics. (b) The active Cdc48 is a ring-shaped hexamer. (c) Cdc48 and its cofactors assemble in a Swiss army knife manner.

 The biochemical basis for broad utility of Cdc48 in cell biology lies in its ATPase activity. ATPase function was first demonstrated with p97, Xenopus homologue of Cdc48 [9], and subsequently shown to be a conserved feature of Cdc48 homologues from yeast to human [5, 7]. Cdc48 belongs to the AAA ATPase family (ATPase associated with diverse cellular activities) that shares common structural organization and often works like chaperones [3, 6, 12, 13]. Cdc48 hexamerizes into a barrel-shaped structure with a central pore (Figures 2(a) and 2(b)). At its core, Cdc48 has two ATPase domains D1 and D2, both of which contain a Walker A and Walker B motif for ATP binding and hydrolysis (Figure 2(a)) [5, 7, 14]. Hexamerization of Cdc48/p97 promoted by ATP binding to D1 domain is required for its ATPase activity and biological function. During ATP hydrolysis, D2 undergoes a major structural change that could generate a pulling force to disassemble a protein complex, while D1 ring remains stable [5, 7, 15]. 

The two ends of Cdc48, the N domain and C tail, are structurally flexible and mainly involved in selecting and/or processing cargoes (Figures 2(a) and 2(b)), but they could also modulate ATPase activity via either posttranslational modification or protein-protein interaction [2, 5, 15]. Through its various cofactors and ATP activity, Cdc48 determines the fates of its substrates, often by extracting the client protein from its binding partners. However, it remains unresolved whether substrates are unfolded on the surface of Cdc48 or by threading through the central pore of Cdc48, like ClpB and GroEL ATPases [5–7, 15]. The precise mechanism underlying the action of Cdc48 in diverse processes awaits further elucidation. 

### 2.2. Ubiquitin Binding

The action of Cdc48 is intimately tied to ubiquitin since nearly all Cdc48-regulated events involve ubiquitin, an evolutionally conserved small protein (~8 kDa) [3–5, 11]. Ubiquitin is often covalently attached, singly or in a chain, to lysine residues on the substrates [16, 17]. Ubiquitin is best known as a molecular flag that marks proteins for destruction by the 26S proteasome but can also lead to changes in protein activity, localization, or conformation [16, 17].

Cdc48 was shown to directly bind ubiquitin via the N domain [18]. Interestingly, the ubiquitin binding was not observed in the presence of ADP, suggesting that a nucleotide-dependent conformational change may be required to expose the binding site on the N domain. However, the specific role and functional significance of the Cdc48-ubiquitin association remain unclear, partly because more focus is placed on many Cdc48 cofactors that exhibit stronger affinity for ubiquitin [5, 11, 19, 20]. Further analysis will be required to distinguish the *in vivo* contribution of ubiquitin-binding activities from Cdc48 and its cofactors in specific cellular events.

Cdc48 regulates nonubiquitin related cellular events as well. Cdc48 contains a putative motif that may recognize Small Ubiquitin-like Modifier (SUMO) [21]. Although the direct binding between Cdc48 and SUMO has yet to be demonstrated, Ufd1, a Cdc48 cofactor, can bind SUMO [21, 22]. Interestingly, Cdc48 was recently shown to regulate SUMO-dependent processes in yeast and human via Ufd1-mediated SUMO-binding [23]. Specifically Cdc48 acts as a chaperone to modulate the association of DNA repair protein Rad51 to DNA upon DNA damage [23].

### 2.3. Association with Diverse Cofactors

Cdc48 serves as the landing pad for its cofactors that confer substrate specificity (Figure 2(a)). If Cdc48 is regarded as a Swiss army knife of cell biology, the Cdc48-interacting cofactors (>40) are then the tools (e.g., blade, corkscrew, hook, etc.) assembled in various combinations (Figure 2(c)), which allow its broad application in biology. The functional diversity of Cdc48 is achieved through its associations with an increasing number of cofactors (e.g., Ufd1-3, SVIP, Png1, and Ubx1-7), many of which also contain ubiquitin-binding motifs that allow simultaneous interactions with Cdc48 and ubiquitylated substrates in myriad cellular pathways [2, 5, 11, 19]. Most of these cofactors possess conserved Cdc48 binding motifs such as UBX (ubiquitin regulatory X), UBX-like element, BS1 sequence, VBM (VCP-binding motif), PUB, and VIM (VCP-interacting motif) [5, 11, 24–26]. Many of these cofactors are not essential for cell growth and survival, suggesting possible functional redundancy among them. Of seven UBX-containing genes identified in *S. cerevisiae*, yeast cells lacking any one of these UBX factors are viable, but deletions of multiple yeast UBX genes lead to severe phenotypes including cell death, suggesting that they have critical, overlapping functions [11, 24, 25].

Most, if not all, of these cofactors are known to hang on the two ends of Cdc48, the N domain and C tail (Figures 2(a) and 2(c)). Based on their biological roles, Cdc48 cofactors are classified into two different functional groups, substrate-recruiting factors and substrate-processing factors [2, 27, 28]. Whereas some of these cofactors (e.g., Ufd1 versus p47, Ufd2 versus Ufd3) compete for the docking site on Cdc48 [5, 19, 27], other cofactors can coexist in the same Cdc48 complex that likely work together to regulate specific cellular events [5, 6, 11, 29, 30]. For example, ubiquitin-binding proteins Ufd1 and p47 assemble distinct Cdc48 complexes. In the complex composed of Cdc48-Ufd1-Npl4-Ubx2, Ubx2, an ER membrane protein is responsible for tethering the complex onto ER; Ufd1 and Npl4 can recognize and present ubiquitin-decorated misfolded secretory proteins for extraction out of the membrane by Cdc48 ATPase [3, 5, 11]. On the other hand, p47 and a deubiquitylating enzyme VCIP135 form another Cdc48 complex that promotes Golgi reassembly in an ubiquitin-dependent, nonproteolytic fashion [31, 32]. Cdc48 also uses its C tail to form distinct complexes with ubiquitin ligase Ufd2, which promotes ubiquitin chain assembly onto the substrates, and an ubiquitin-binding protein Ufd3, which works with a deubiquitylation enzyme Otu1 to inhibit ubiquitin chain synthesis [27, 33, 34]. These actions suggest that Cdc48 provides a platform for two opposing activities (i.e., ubiquitylation and deubiquitylation) and thereby facilitating tight temporal and spatial control over the ubiquitylation status/fates of substrates [5, 35]. 

Since many Cdc48 cofactors have been identified and are involved in diverse processes (Table 1), it is imperative to sort out which Cdc48 cofactors coexist in one complex or are incompatible with each other and further assign specific function to each complex. Among Cdc48 cofactors, some of them known to exist in one complex are Ufd1-Npl4 and gp78 ubiquitin ligase, Png1 glycanase and gp78, FAF1 and Ufd1-Npl4, Derlin and Ufd1, and Ufd1 together with Ubx2 and Ufd2. The Cdc48 cofactors that appear to be incompatible with each other include Ufd1 and p47, Ufd2 and Ufd3, Ufd1 and Vms1, SVIP and p47, SVIP and Ufd1, p37 and Ufd1, and gp78 and SVIP [2–6, 10, 11]. 

## 3. Regulation of Cdc48 through Posttranslational Modifications

Despite its prevalence in cell biology, how Cdc48 activity is regulated remains poorly understood. Posttranslational modifications, such as phosphorylation and acetylation, are common mechanisms employed for controlling the way a protein behaves inside cells. For instance, the localization, enzyme activity, stability, and structure of a protein could be changed upon these modifications in response to internal or external challenges. Phosphorylation has been demonstrated to modulate the function of Cdc48 in several ways. Cdc48 can be phosphorylated at several tyrosine residues. Mammalian Cdc48 was first found to be phosphorylated at tyrosine residues 796 and 805 upon T-cell activation without affecting ATPase activity [36, 37]. Phosphorylation of corresponding residue of Tyr^805^ in yeast Cdc48 leads to structure alteration that allows the exposure of the N-terminal nuclear signal, which triggers subsequent nuclear import of Cdc48 in late G1 phase of the cell cycle [38]. Phosphorylation of Tyr^805^, likely by v-Src kinase, completely eliminates the interaction between Cdc48 and Ufd3 or PNGase [29, 39], which is involved in degradation of misfolded secretory proteins [40], further suggesting a regulatory role of phosphorylation in Cdc48's proteolytic function [39]. The action of Cdc48 in transitional ER assembly appears to be modulated by Jak2 kinase-mediated Tyr phosphorylation [41]. Dephosphorylation of Cdc48 catalyzed by PTPH1 phosphatase stabilizes the Cdc48-ER membrane association, thereby promoting ER transitional assembly [41]. Furthermore, in human U937 myeloid leukemia cells, a Tyr phosphorylated Cdc48 species is preferentially accumulated in the cytosol upon release from growth arrest, suggesting a possible role of Cdc48 in leukemic differentiation process [42]. 

Phosphorylation on serine and threonine residues could also regulate Cdc48 function [43]. Upon DNA damage, Cdc48 is phosphorylated at Ser^784^ by DNA-PK and accumulates at sites of DNA lesion [44], suggesting that Ser^784^ may be the key to DNA damage-triggered signaling. Under sustained hypoxia in PC-12 cells, Akt kinase can phosphorylate Cdc48 on Ser^352^, Ser^746^, and Ser^748^ [45], which leads to markedly reduced association between Cdc48 and ubiquitylated protein. Furthermore, the phosphomimetic form of Cdc48 on Thr^761^ exhibits elevated ATPase activity [46]. 

Besides phosphorylation, acetylation has been detected at several sites of Cdc48 and can affect its ATPase activity [43, 46]. Cdc48 was also shown to be S-nitrosylated at three sites (i.e., Cys110, Cys526, and Cys664) or methylated at Lys315, which inhibit its ATPase activity [47, 48]. Cdc48 may be decorated with SUMO [49] and ubiquitin [50]. However, the biological functions and significance of these modifications remain to be established. Unraveling the structural and functional consequences of these modifications on Cdc48 will be an important step in elucidating the mechanisms that allow the multifunctional usages of Cdc48.

## 4. Biological Functions

Cdc48 plays essential roles in cell growth and survival as demonstrated by phenotypes associated with yeast *CDC48* mutants and targeted deletion of mouse Cdc48 [1, 6, 51]. Consistent with its broad cellular distribution and abundance, Cdc48 regulates a myriad of physiological events (Table 1), which have been recently covered by excellent reviews [3–5, 10, 11]. For example, Drs. Dantuma and Hoppe discussed growing evidence and recognition that Cdc48 can act as a segregase in ubiquitin-dependent, but nonproteolytic fashion [3], supported by the reports showing that ubiquitin attachment onto ER-localized Spt23 [52], nuclear transcription factor Mata2 [53], or RNA polymerase subunit Rpb1 [54] attracts Cdc48 to release them from their sites (e.g., ER membrane, chromatin, and binding partner), which is pivotal for gene expression or DNA repair. Here we focus on three Cdc48-regulated processes, the Ubiquitin Fusion Degradation (UFD) pathway that was the first proteolytic route linked to Cdc48, endoplasmic reticulum associated degradation (ERAD) in which Cdc48's function is probably best characterized, and autophagy in which an understanding of Cdc48's involvement is just emerging.

### 4.1. The Ubiquitin Fusion Degradation (UFD) Pathway

The first indication that Cdc48 promotes substrate proteolysis came from its interaction with Ufd3 [66], which was initially isolated in a screen for genes that were required for the turnover of artificially designed UFD substrates (e.g., Ub-Pro-*β*gal, Ub^V76^-Val-*β*gal, and Ub^V76^-Val-DHFR), in which the N-terminal appendage of ubiquitin targets the reporter proteins (e.g., *β*gal, DHFR) for destruction by the ubiquitin-proteasome system [67]. Due to the scarcity of *in vivo* substrates of the ubiquitin pathway in earlier days, the studies of synthetic substrates, such as UFD and N-end rule substrates, were instrumental in uncovering many key mechanistic insights about ubiquitin-mediated proteolysis [68, 69]. The genetic dissection of the involved degradation pathways illuminated many ubiquitylation and postubiquitylation events [67–69], including the isolation of first E2 and E3 enzymes (i.e. Rad6 and Ubr1) and the first E4 enzyme (i.e., Ufd2) [70]. The early evidence for the involvement of Ufd1, Rad23, Dsk2, Cdc31, and Rad4 in proteolysis came from the work on the UFD pathway [66, 67, 71]. Importantly, these factors were later shown to be involved in many crucial cellular processes *in vivo*, and physiological UFD substrates (e.g., Ubb+1) have been identified, validating the employment of these model substrates [68, 69, 72, 73]. 

Cdc48 plays several distinct roles in the UFD pathway. One function of Cdc48 is to promote substrate ubiquitylation [34, 70, 74]. UFD substrates (e.g., Ub^V76^-Val-*β*gal) are first decorated with a few ubiquitin molecules, which is insufficient to trigger degradation, by Ufd4 E3 ligase along with ubiquitin-activating and -conjugating enzymes (i.e., E1, E2) [70]. Oligoubiquitylated UFD substrates are then recognized by the Cdc48-Ufd1-Npl4 complex, which brings along Ufd2, a ubiquitin chain elongation factor (E4), to specifically promote ubiquitin chain extension onto UFD substrates (see the model in [74]). This assistance of Cdc48 on Ufd2's substrate recognition is supported by the disruption of *in vivo* Ufd2-substrate association in temperature sensitive cells bearing *npl4* or *ufd1 *mutations [74]. The requirement of Cdc48's ATPase activity for this step remains to be determined. 

Another function of Cdc48 is after ubiquitylation to promote subsequent transfer of ubiquitylated UFD substrates to the proteasome for destruction. *In vivo* studies suggest that Cdc48 may also act downstream of the ubiquitylation reaction by Ufd2, as stabilized UFD substrates are ubiquitylated in *cdc48* and *ufd1* mutants [34, 70]. One postubiquitylation event that is under the control of Cdc48 is the interaction between Ufd2 and Rad23 [34]. With its abilities to bind to ubiquitin chain and the proteasome using two separate domains (i.e., UBA, UBL), Rad23 is a key molecule in bringing ubiquitylated proteins to the proteasome [16]. Rad23 is recruited to the ubiquitylation machinery via its binding to Ufd2, which in turn facilitates target recognition by the UBA domain of Rad23 (see the model in [34]). Through its association with Ufd2, Cdc48 uses its ATPase activity to promote the disengagement of Ufd2 and Rad23, which allows the release of substrate loaded Rad23 from Ufd2 and thereby facilitating an orderly handoff of the substrate from the ubiquitylation machinery to the proteasome [34]. 

Studies of several synthetic UFD substrates (e.g., Ub^V76^-GFP, Ub^V76^-DHFR) also revealed that Cdc48 has other Ufd2-independent role(s) in the UFD pathway. The Cdc48-Ufd1-Npl4 complex is proposed to use its ATPase activity to unfold tightly packed substrates, which may be challenging and time consuming for proteasome processing. Requirement of Cdc48 can be relieved for well-folded UFD substrates if they contain a sufficiently long (>20 amino acids), flexible element [75], which is necessary for subsequent proteasome engagement. 

Although Cdc48 was first found to associate with Ufd3 [66], the specific role of this interaction in the UFD pathway remains unclear. The original isolation of *ufd3* mutant in the screen for UFD regulators was due to the depletion of free ubiquitin in cells lacking *UFD3*, which affects global proteolysis and could be rescued by enhanced ubiquitin expression. Ufd3 also cooperates with a deubiquitylating enzyme Otu1 and competes with Ufd2 for Cdc48 binding, which antagonizes Ufd2-catalyzed ubiquitylation reaction [27]. 

### 4.2. Endoplasmic Reticulum Associated Degradation (ERAD)

The most extensively characterized function of Cdc48 is its pivotal roles in ERAD [5, 76, 77], a protein quality control process, the significance of which has been increasingly appreciated owing to its emerging, prominent role in human diseases including cancer, Alzheimer's disease, diabetes, and lung emphysema. About one-third of cellular proteins travel through the ER, the folding state of secretory proteins is under stringent surveillance in the ER to ensure their quality [5, 77]. Only properly folded proteins move on to their destination to carry out their cellular functions. Immature proteins are retained in the ER for folding by ER chaperones. To prevent deleterious effects of the accumulation/aggregation of aberrant proteins, terminally misfolded proteins are destroyed via ERAD. More specifically, these unwanted proteins are ejected to the cytosol by an unknown retro-translocation mechanism. Then, the substrates are recognized by ubiquitin ligases that tag the clients with ubiquitin molecules. The ubiquitylated substrates are subsequently escorted to and degraded by the proteasome. ERAD substrates include HMG-CoA reductase, T-cell receptor *α* chain, antitrypsin, cystic fibrosis transmembrane regulator, and apolipoprotein B [5, 77]. 

Based on the location of the misfolded domain (e.g., membrane, lumen, or cytosol) and the topology of the protein, ERAD substrates are sorted into different degradation pathways defined by distinct ubiquitin ligases (e.g., Hrd1, Doa10, and gp78) [5, 77–79]. And yet these pathways seem to converge on Cdc48 for the final leg of the journey to the proteasome [5, 77]. Like its involvement in the UFD pathway, Cdc48 exerts power over multiple events in ERAD at stages pre- and postsubstrate ubiquitylation. 

Cytosolic Cdc48 is tethered to the ER through the tight association with its cofactors (e.g., Ubx2, VIMP) embedded in the ER membrane, which facilitates the incorporation of Cdc48 into the protein network designed for highly coordinated ERAD [5, 77, 80]. Two Cdc48 cofactors gp78 and Ufd2 are ubiquitin ligases, which decorate their cargoes with ubiquitin and are part of this ensemble that selects misfolded ER proteins for destruction by the proteasome.

Cdc48 works on both nonubiquitylated and ubiquitylated ERAD targets [20, 81]. In *cdc48* mutant, ERAD substrates are not ubiquitylated [81], suggesting that Cdc48 may be required for ubiquitylation albeit the precise function of Cdc48 remains elusive. As misfolded proteins emerge from the ER, they are recognized by ER resident E3 ubiquitin ligases such as gp78, Doa10, and Hrd1. Cdc48 binds to these ERAD substrates [20, 82]. With its ability to differentiate the native versus nonnative state of a protein [83], Cdc48 could act as a chaperone in holding misfolded substrates exposed to an entirely new environment (i.e., cytosol), which in turn could prevent substrate backtrack and/or protein aggregation, in a state ready for ubiquitylation and degradation. 

Unlike many degradation targets, ubiquitylated ERAD substrates then face a physical barrier (i.e., the ER membrane) that substrates need to traverse to reach the proteasome in the cytosol. Cdc48 ATPase plays an essential role in the dislodgement of misfolded proteins from the ER membrane by providing the power for the energy-demanding substrate retrotranslocation [5, 76, 77]. ERAD substrates remained in the ER membrane in *cdc48 *mutants [81]. Armed with two ubiquitin binding cofactors Npl4 and Ufd1, Cdc48 is locked onto ubiquitylated cargoes [5, 19]. The ubiquitin binding may also send a signal to Cdc48 to pull substrates out of the ER, which requires the energy derived from ATP hydrolysis by Cdc48 [5, 77, 81].

Substrates extracted by Cdc48 may take different routes to the proteasome. The precise understanding of underlying pathway selection remains murky but is likely dependent on different features such as ubiquitin chain length/linkage involved, folding status, cargo size, and the presence of sugar chains. For example, a short ubiquitin chain assembled on substrates may be further extended by Cdc48 associated with Ufd2 ubiquitin chain elongation factor E4 [70, 74], which then shuttles cargoes to the proteasome through the Ufd2-Rad23 connection [40, 74, 84]. Some glycoproteins may first need to be stripped off their sugar chains, which could be too bulky for the entry of the proteasome, by N-glycanase Png1, another Cdc48 cofactor [29, 40]. Some substrates may be quickly loaded onto the proteasome that is closely tied to Cdc48 or the ER membrane [5]. Cdc48 is also found to associate with deubiquitinating enzymes (e.g., Otu1), which may rescue or remodel some substrates through ubiquitin chain editing [27, 35]. 

Although clearly Cdc48 is central in coordinating retrotranslocation, ubiquitylation, and degradation of ERAD substrates, our understanding of Cdc48's precise role in conducting this cellular symphony remains sketchy. For example, how does Cdc48 partner with these many distinct cofactors? Do these cofactors cycle on and off Cdc48 or do different Cdc48 ensembles form a relay? How do Cdc48 and its cofactors respond to different cellular signals? How does Cdc48 work with a variety of proteins to coordinate various upstream and downstream events together? 

### 4.3. Autophagy

The discovery of Cdc48 as a causative factor for Paget's disease of bone [85, 86], a degenerative disorder associated with compromised autophagy, first revealed Cdc48 as a key regulator of autophagy, another major proteolytic system in eukaryotes [87, 88]. Autophagy has historically been deemed as a nonspecific degradation process that eliminates proteins and organelles in bulk by the lysosome to provide cellular nutrients in time of stresses (e.g., starvation), but it is emerging as a regulatory system that selectively destroys specific proteins such as *α*1-antitrypsin, APP/*β*-amyloid, and Huntingtin, the accumulation of which would be detrimental to cells [87–89]. 

The common feature shared by various types of autophagy is that cytoplasmic cargoes are engulfed by the autophagosome, a double-membrane vesicle structure, and then escorted to and fused with the lysosome for elimination by resident hydrolases [88, 89]. Over 30 autophagy-related (ATG) genes have been reported, and 15 of these are “core” components commonly required for distinct autophagic pathways. In response to different environmental stimuli, various adaptations of the core autophagy machinery allow cells to accordingly modulate cellular contents, including organelles (peroxisome, ribosome, and mitochondria) or intracellular proteins (e.g., Huntingtin, *α*-synuclein) [88, 89].

Although only a little is known about the involvement of Cdc48 in autophagy, it appears that Cdc48 regulates multiple autophagy events, including maturation of autophagosomes, inclusion body formation, as evident by the accumulation of immature autophagic vesicles, and inclusion body myopathy in cells bearing Cdc48 pathogenic mutants [90, 91]. Cdc48 is tied to pathologic protein inclusions in several diseases associated with compromised autophagy activity, such as Lewy bodies in Parkinson disease, SOD positive inclusions in amyotrophic lateral sclerosis, and Huntingtin inclusion in Huntington disease [4, 91, 92]. Consistent with its multiple functions, Cdc48 interacts with several autophagy regulators, including a core factor Atg8/LC3, HDAC6 deacetylase, and regulators of specific autophagy branches such as the Ubp3-Bre5 deubiquitylation complex in ribophagy [4].

Cdc48 is crucial to autophagosome biogenesis under starvation conditions through the direct interaction between its cofactor Ubx1 and a ubiquitin-like molecule Atg8/LC3 that is conjugated to phosphatidylethanolamine, the lipid enriched in the autophagic membranes [93]. *ATG8* mutants are defective in autophagosome formation and membrane expansion. Ubx1 preferentially binds lipidation induced Atg8 oligomers. The precise function of the Ubx1-Atg8 interaction remains unclear, but it is proposed to allow Cdc48 to extract Atg8 out of autophagosome membrane, which in turn promotes autophagosome enclosure. It is yet to be determined whether the ubiquitin binding and ATPase activity are essential for Cdc48's action in this process.

Cdc48 is required for ribophagy, an autophagy branch that selects ribosomal components for destruction by the lysosome in response to nutrient crisis [4, 94]. Specifically, Cdc48 and its cofactor Ufd3 have been shown to work with the Ubp3-Bre5 deubiquitylating complex to promote the lysosome-mediated turnover of Rpl25 protein. *RPL25* encodes a ribosomal subunit, which is rapidly degraded by autophagy upon starvation to allow cells to tune down protein synthesis. 

Cdc48 and Ufd3 have been shown to interact with another autophagy regulator HDAC6, a cytosolic deacetylase [95–97]. HDAC6 binds to polyubiquitylated aggregates via C-term ubiquitin binding BUZ domain and dynactin, a component of the dynein motor complex [98–100]. HDAC6 is thought to bring ubiquitylated substrates to inclusion bodies localized around the microtubule-organizing center via the microtubular transport system, and thereby promoting cargo transport to the autophagosome for subsequent destruction by autophagy [97]. The Cdc48-HDAC6 interaction has been proposed to facilitate the loading of cargos onto the HDAC6-dynein complex [96, 97]. 

Cdc48 also regulates mitophagy, an autophagy-mediated destruction of dysfunctional mitochondria. The mitophagy involvement of Cdc48 is deemed to be indirect so far since Cdc48 is required for proteasome-mediated degradation of two GTPases Mfn1 and Mfn2 mitofusin that mediate mitochondria fusion [101, 102]. Many tantalizing links between Cdc48 and mitophagy players (e.g., Parkin) exist and remain to be dissected [4, 99]. 

## 5. Diseases Caused by Cdc48 Mutations

Involvement of Cdc48 in myriad cellular processes suggests that it may be important for human health and disease [4, 6, 103]. Indeed, consistent with its role in genome maintenance and modulation of various oncogenes and tumor suppressors (e.g., HIF1a, p53, I*κ*B*α*, Brca1, NF*κ*B, and NF1), Cdc48 expression and function have been tied to human malignancies including cancers of breast, liver, lung, pancreas, ovary, and colon [6, 103]. More importantly, mutations in Cdc48 have been linked directly to neurodegenerative diseases including inclusion body myopathy associated with Paget's disease of the bone and frontotemporal dementia (IBMPFD) and amyotrophic lateral sclerosis (ALS) [85, 86, 104]. 

The common pathogenic characteristics in autosomal-dominant IBMPFD are the ubiquitylated inclusions in muscle, brain, and bone tissue that cause muscular dystrophy, neurodegeneration, and frequent bone fracture [55, 90]. Currently 27 missense mutations in Cdc48 gene have been isolated from IBMPFD (e.g., I27V, R93C, R95C, R95G, G97E, P137L, R155C, R155S, R155L, R155P, G157R, R159H, R159C, L198W, I206F, A232E, T262A, N387H, A439S, and A439P) and/or ALS (R95H, I151V, R155H, R159G, R191G, R191Q, and D592N) patients worldwide including countries like China, Japan, Argentina, USA, Germany, Italy, and Australia [90, 105–108]. The majority of mutations are located on or closed to the N and D1 domains of Cdc48 and are impaired for autophagy and the degradation of ERAD substrates (e.g., CFTR) or other proteasomal targets (e.g., UNC-45). Other molecular defects found in IBMPFD patients include mitochondrial dysfunction, the accumulation of ubiquitylated species, and TAR DNA binding protein-43 (TDP-43), a major disease protein in frontotemporal dementia and ALS. Although IBMPFD associated mutations in Cdc48 often retain a normal hexameric oligomer, some mutations affect its ATPase activity and bindings to cofactors and/or substrates [56, 58–62]. Mutations in Cdc48 and associated biochemical defects that have been dissected are listed in Table 2. The molecular basis for IBMPFD and ALS caused by these Cdc48 mutations remains elusive. Distinct clinical features exist among different IBMPFD patients. The key to unravel the pathogenesis of these mutations is to comprehensively analyze their effects on cofactors bindings and specific substrate degradation.

## 6. Perspective

The field of ubiquitin and ubiquitin binding proteins has been evolving rapidly in recent years. Cdc48 plays a central and dynamic role in many ubiquitin-mediated events. Despite general realization of and interests in wide-ranging functions of Cdc48, we have only scratched the surface of its biological roles as evident by recent exciting discoveries of Cdc48's involvement in SUMO-dependent events [23] and in the ribosome-associated degradation (RAD) that eliminates misfolded nascent peptides [109, 110], which may include up to ~30% of newly synthesized proteins. For most of the cofactors identified so far, the physiological role and significance of their interaction with Cdc48 are yet to be clearly defined [3–5, 11]. Although Cdc48 can bind ubiquitin on its own, how this activity contributes to a specific process is little known with more focus having been placed on the ubiquitin binding properties of Cdc48 cofactors. Also poorly characterized is Cdc48's binding to lipids [111], which may be critical to its function because Cdc48 is often attracted to various membranes (e.g., ER, autophagosome, Golgi, and mitochondria) decorated with lipids. Interestingly, like its archaeal counterpart, Cdc48 contains an HbYX motif that could be docked onto the core particle of the proteasome, raising an intriguing possibility that Cdc48 may cap on one end of the 20S core proteasome particle and recognize and thread substrates into the proteolytic chamber for destruction [112, 113], which may be challenging to 19S regulatory particles. 

In spite of recent advances, we have much to learn about Cdc48. For example, how does Cdc48 juggle so many pivotal cellular events under normal and various stress conditions? Does Cdc48 participate in nonubiquitin and/or non-ATPase dependent reactions? How does Cdc48 coordinate two major cellular degradation systems, the proteasome, and autophagy? How many more cofactors exist for Cdc48, and how are these cofactors organized into the same or different complex? Does Cdc48 modulate its own complex assembly and disassembly in response to internal and external stimuli? What are the specific functions and substrates of each Cdc48 complex? Could mutations in Cdc48 directly lead to cancer? How does Cdc48 and its cofactors work with other ubiquitin binding proteins (e.g., Rad23, Rpn10, p62, and Nbr1) that are involved in proteasome or autophagy-mediated pathways? Understanding these issues will undoubtedly illuminate many areas of cell biology.

The work on Cdc48 may also bring about potential therapeutic strategies for Cdc48-related diseases. The components of the ubiquitin/proteasome system are attractive drug targets, as illustrated by the efficacy of some proteasome inhibitors in the treatment of multiple myeloma and other cancers [17, 68]. Indeed, specific Cdc48 inhibitors have been developed [114, 115]. For instance, small molecule DBeQ (N2, N4-dibenzylquinazoline-2,4-diamine) efficiently blocks multiple Cdc48-regulated processes, including autophagy and proteasome-mediated degradation of UFD and ERAD substrates [116]. Furthermore, DBeQ rapidly activates apoptotic cell death and inhibits cancer cell growth [115, 116]. Other potent Cdc48 inhibitors include Eeyarestatin I [114], alkylsufanyl-1,2,4-triazoles, and sorafenib [115, 117]. Similar to proteasome inhibitor Bortezomib, these inhibitors exhibit anticancer activity. Since Cdc48 regulates a subset of proteolysis, Cdc48 related drugs may have less side effects than the proteasome inhibitors. With its ever-expanding utility, the stock of Cdc48 is sure to continue its rise in the coming years.

## Figures and Tables

**Table 1:** Functions and substrates of some Cdc48 cofactors.

Cofactor	Function	Substrates
Ufd1-Npl4	ERAD, cell cycle control, nuclear transport, DNA repair, transcription, ribosome-associated degradation, essential for cell growth, clearance of damaged mitochondria	**Spt23**, **Mga2**, **Sec23**, **Mps1**, **Ole1**, **Hmg2**, CPY*, CT*, CTG*, Ste6*, **Pex29**, **Rad52**, **Rpb1**, **Mat*α*2**, **Gnd1^PTC^**, **GFH^NS^**, **Fbp1**, **UFD model substrates**, Ci/Gli3, CD3*δ*, CD4, TCR*α*, HO-1, CDT1, SET8, tyrosinase mutant, L3MBTL1, SKP2, MHC class I heavy chains, inositol 1, 4,5-triphosphate receptors, Hmg-CoA reductase…

Ufd2/E4B	Ubiquitin chain synthesis, cell cycle control, ERAD, cardiac development, and nervous system maintenance	**Mps1**, **Pex29**, Ste6*, **Hmg2**, **Spt23**, **Fad3**, **UFD model substrates**, p53, p73, Ataxin-3, Fez1, myosin chaperone UNC-45

Ufd3/PLAA	Ubiquitin homeostasis, ribophagy, tRNA import to mitochondria, DNA repair, multivesicular body sorting	**Cse4**, **Rpl25**, **Spt23**, **Cps1**, **Vph1**, **Fad3**, **UFD model substrates**, **PCNA**, **histone H2B**, **N-end rule**

Vms1/ANKZF-1	ERAD, mitochondria stress response	**Cdc13**, **Fzo1**, **CFTR**, CPY*

Ubx1/p47/Shp1	Autophagy, nuclear envelop formation, ER biogenesis, Golgi assembly	**GFP-Osh1**, **Fad3**, **UFD model substrates**, NEMO, Rum1

Ubx2	ERAD, lipid droplet maintenance	**Hmg2**, CPY*, **KSS**, Ubc6*, **Sec61-2**, **Deg1-GFP**, **Spt23**, SREBP, RNA binding protein HuR, Insig-1, ApoB-100, UBC6-B5 mutant

Ubx3	*Unknown *	*Unknown *

Ubx4	ERAD, DNA repair	CPY*, Ste6*, **Fbp1**, **Rpb1**

Ubx5	Links Cdc48 to CRL E3 ligases	**Rpb1**, HIF1*α*

Ubx6	*Unknown *	*Unknown *

Ubx7	*Unknown, in complex with Dfm1 *	*Unknown *

FAF1	ERAD, proapoptosis factor	CD3*δ*, Hsp70, *β*-catenin

Ataxin-3	Ubiquitin chain editing, ERAD, stress response	TCR*α*, BACE mutant, SOD1, PARKIN, CHIP, integrin subunits, UFD model substrates

Substrates of Cdc48 cofactors in yeast *S. cerevisiae* are indicated in bold.

**Table 2:** Mutations and defects in Cdc48-related diseases.

Mutation	Domain located	Disease	Biochemical defects
R93C	N domain	IBMPFD	Impaired degradation of ERAD substrates (e.g., CFTR, tyrosinase) [55, 56]; restored the growth of yeast cdc48 mutant at 37°C [57].

R95G	N domain	IBMPFD	Increased bindings to Ufd1, Npl4, p47, Ataxin 3, and ubiquitylated substrates; reduced bindings to Ufd2, CAV1, and UBXD1; little effects on the bindings to Hrd1, Png1 [58–60]; altered response to nucleotide-triggered conformation change of the N domain [61, 62]; enhanced ATPase activity and polyQ aggregation; compromised degradation of the myosin chaperone UNC-45 and ERAD substrates [63, 64]; impaired proteasome activity; accumulation of ubiquitin conjugates and TDP-43; induced cell death [6, 63, 64].

P137L	N domain	IBMPFD	Abolished bindings to Ufd1, Npl4, and p47, but still bind gp78 [60]; reduced solubility and altered cellular localization; impaired ERAD [60, 64].

R155H The most prevalent mutation	N domain	IBMPFD and ALS	Increased bindings to Ufd1, Npl4, p47, Ataxin 3, and ubiquitylated substrates; reduced bindings to Ufd2, CAV1, UBXD1; little effects on the bindings to Hrd1, Png1 [58–60]; altered response to nucleotide-triggered conformation change of the N domain [61, 62]; enhanced ATPase activity and polyQ aggregation; compromised degradation of the myosin chaperone UNC-45; impaired ERAD, autophagy, and proteasome; accumulation of ubiquitin conjugates and TDP-43; induced cell death; mitochondria defects [6, 63–65].

R155S	N domain	IBMPFD	Enhanced bindings to Ufd1, Npl4, p47, and Ataxin 3 [59].

R155C	N domain	IBMPFD and ALS	Increased bindings to Ufd1, Npl4, p47, Ataxin 3, and ubiquitylated substrates [59]; enhanced ATPase activity and polyQ aggregation; impaired ERAD and proteasome; accumulation of ubiquitin conjugates and TDP-43; induced cell death; mitochondria defects [63–65]; rescued yeast cdc48 mutant [57].

R155P	N domain	IBMPFD	Increased bindings to Ufd1, Npl4, p47, and ubiquitylated substrates [59]; enhanced ATPase activity and polyQ aggregation; normal hexmer formation; altered conformation of the D2 ring; compromised degradation of ERAD substrates [61–63]; rescued yeast cdc48 mutant [57].

R159G	N domain	ALS	Compromised degradation of ERAD substrates [55, 56, 63].

R191Q	N-D1 linker	ALS and IBMPFD	Increased bindings to Ufd1, Npl4, p47, and ubiquitylated substrates [59]; enhanced ATPase activity and polyQ aggregation; altered response to nucleotide-triggered conformation change of the N domain [61, 62]; compromised ERAD and proteasome; accumulation of TDP-43; induced cell death [63, 64]; mitochondria defects [65]; rescued yeast cdc48 mutant [57].

L198W	N-D1 linker	IBMPFD	Enhanced binding to ubiquitylated substrates; impaired ERAD [59, 60, 63].

A232E	D1 domain	IBMPFD and ALS	Increased bindings to Ufd1, Npl4, p47, and ubiquitylated substrates; reduced bindings to CAV1, UBXD1 [58, 59]; normal hexmer; altered response to nucleotide-triggered conformation change of the N domain; altered conformation of the D2 ring [61, 62]; enhanced ATPase activity and polyQ aggregation; impaired ERAD, autophagy, and proteasome; accumulation of ubiquitin conjugates and TDP-43; induced cell death [63]; rescued yeast cdc48 mutant [57].

T262A	D1 domain	IBMPFD	Impaired degradation of ERAD substrates [55, 56, 63].

N387H	D1 domain	IBMPFD	Compromised ERAD; accumulation of ubiquitin containing inclusion [55, 63].

I27V, R95C, R97C, R97E, R155L, G157R, R159H, R159C	N domain	IBMPFD	

R95H, I151V	N domain	ALS	

I206F	N-D1 linker	IBMPFD	

R191G	N-D1 linker	ALS	

A439S, A439P	D1 domain	IBMPFD	

D592N	D2 domain	ALS	
